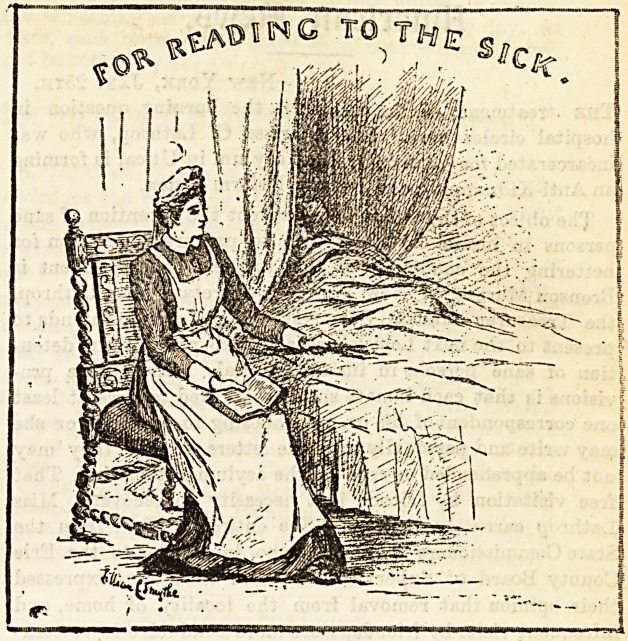# Extra Supplement—The Nursing Mirror

**Published:** 1891-02-21

**Authors:** 


### "The Hospital" Nursing Mirror.

Being the Extra Nursing Supplement of "The Hospital" Newspaper.

Contributions for this Supplement should be addressed to the Editor, The Hospital, 140, Strand, London, W.C., and should have the word "Nursing" plainly written in left-hand top corner of the envelope.

### En Passant.

Southend Institute.—Sister Rose, who was for some time in charge of the sick wards of the Foundling Hospital, has started an Institution and Home for Invalids at Southend-on-Sea. Sister works amongst poor as well as the rich, and is responsible for the district nursing in All Saints' parish, paying 172 visits last month.

Out of Trouble.—Mrs. Berry, who was charged with manslaughter, has been acquitted. The medical men were cross-examined with the view of showing that the puerperal fever might have been engendered by bad drainage, and that the deceased and her husband had only gone into the house at Tooting three weeks before her death. The jury, after hearing this evidence, stopped the case, and acquitted the prisoner. Mr. Justice Charles said he quite agreed with the verdict, but he thought the case was very properly brought before the Court.

Leeds District Nurses.—Sir James Kitson presided at the annual meeting of the Society for the District Nursing of the Sick Poor of Leeds on February 4th. There was a large attendance, many ladies being present. Mrs. Garlick read the report, which stated that the number of cases attended in the past year had been 846, and the visits paid numbered 19,160. These figures, though showing a large amount of work done, were not so high as in the previous year, the reason being that the Committee, finding the nurses were suffering from the continued strain of overwork, decided to curtail each district by cutting off the outlying portion, so making them more workable. The effect on the nurses had been beneficial, and their health had been fairly maintained, but the change was not made without causing disappointment to many. There is room in the home for another nurse, but the funds of the Society are very low; there are now seven nurses on the staff. Dr. Wardrop Griffith spoke highly of the work done by the nurses.

Workhouse Infirmaries.—For the year 1890 the Workhouse Infirmary Nursing Association issues a good report. The Duchess of Westminster has consented to act as President of the newly-formed northern branch. During the year fourteen probationers completed their training, and received appointments. Thirty-four entered for general training, and four trained nurses completed their midwifery course at the expense of the Association. Insufficient night-nursing and the whited-sepulchre state of the country infirmaries are the two giants against which the Association is now fighting. "There is a certain uniformity of aspect in many of the country infirmaries, spotlessly clean wards, methodical arrangement, a minimum of comfort and a maximum of officialism. As to anything approaching to a comfortable seat, that is rarely ever thought of, and as to providing any interests or recreations, that is but little planned for, except where the visits of ladies are allowed." In these excellent infirmaries the Inspector seeks in vain for baths, decent lavatory arrangements, or proper ventilation. It is particularly to be desired that there should be more lady guardians, and that they should be taught to look below the surface. If this were done, and the Association had more support, a remarkable improvement would be seen. Remember, we are not pleading for the able-bodied pauper, but for the sick.

Short Items.—Nurse Grey, of Lymm, will in future continue her good work amongst the poor under a committee of ladies.—Reels and strathpeys will be danced on March 24th, in aid of the Edinburgh Jubilee Institute. Such are the contrasts of life.—Miss Rotherham and Miss Jack have been lecturing to the Edinburgh medical students on "Peptonised Foods."—During the absence of the nurse a patient in the Hope Hospital jumped out of the window, and was killed. Why was the nurse absent?—At an inquest on a late patient of Northwich Infirmary, the jury wanted to question a nurse, who was giving evidence, in face of the objection of the Coroner. It would be much better to permit more questions when the jury are not fully satisfied.

A New Home.—Liverpool, ever to the fore in nursing matters, can now boast a Home in connection with the Southern Hospital, and the new Home was blessed by telegram by Florence Nightingale. We hope that bit of pink paper will be framed, and hung in the nurses' sitting-room. The Home contains room for forty nurses, but there is still a debt of £1,000 on the building fund. The Home was opened by Mrs. Horsfall with a beautifully-designed key. The following is the notable telegram: "God speed the Nurses' Home to be opened to day; and all its dear nurses and probationers and Matron and home sister. God bless their quiet, steady, loving progress towards the best, year after year; and God guide their good President and Committee and officers is the fervent prayer of Florence Nightingale."

Infirmary Matters.—Croydon Guardians have received thirty-five applications for the post of Lady Superintendent. They propose to appoint a probationer, in which case they must undoubtedly choose a trained Lady Superintendent. It is quaint to hear Guardians talk, as they often do, of having probationers when they have no-one in charge to do the training. At Kingston there is the usual unsatisfactory state of affairs; the Master complaining of the nurses and the nurses complaining of the Master. Of course there ought to be a superintendent over the nurses, and the Master ought to have nothing to do with them. The mere tone in which he speaks of the nurses is enough to show why there is war. Nurse Smith is leaving Richmond Union.

Canterbury Institute.—The report of the Kent and Canterbury Institute for Trained Nurses opens with expressions of regret for the death of Bishop Parry and Dr. Lochée, two firm friends of the movement from the start. The work of the Home has increased, but it has found the usual difficulty in procuring enough nurses, and has now decided to train six annually. "The Committee wish to put on record their high appreciation of the energy and self-sacrifice of the Lady Superintendent, in providing for the charitable work during the vacancy in the post of district nurse, and of her skilful management in arranging with an inadequate staff, to supply nurses for the cases in which application was made for them." The number of nurses on the staff (exclusive of the district nurse) at the beginning of the year was 14; and at the end of the year is nine. They have had charge of 196 cases, and have been almost incessantly employed. It has been found that nurses break their three years' engagement to the Home, so they are in future to be heavily fined if found guilty in this respect.

### Lectures on Surgical Ward Work and Nursing.

By Alexander Miles, M.B. (Edin.), C.M., F.R.C.S.E.

### Lecture XIV.—LIGATURES AND TOURNIQUETS.

Ligatures are used to tie blood vessels; sutures to stitch up wounds. Various materials are in general use for both purposes, silk, catgut, and kangaroo-tendon being chiefly used as ligatures; whale-gut, horsehair, and silver wire as sutures.

(*a*) Catgut has the great advantage over some other forms of ligature of being absorbed by the tissues, so obviating the necessity of its removal by the surgeon, or by ulceration. It is made from the intestine of the sheep, which is first scraped so as to leave only the sub-mucous layer, and then dried and cut into strips of appropriate length and breadth. It is then antisepticised by means of chromic or carbolic acid, and kept either in carbolic and glycerine (1 in 10), or in eucalyptus oil. It is often not reliable as to its asepticity, but can be rendered so by being boiled in a 97 per cent. solution of absolute alcohol for one hour. It has been shown that catgut so treated may be kept in a putrescible fluid for weeks without putrefaction taking place. Always test a catgut ligature before handing it up to the surgeon, to make sure that it will stand the strain put upon it in tying the vessel.

(*b*) Whale-gut has the same advantages as catgut, and is largely used as ligatures and sutures.

(*c*) Kangaroo-tendon is prepared from the strong tendon of the tail of that animal, and has the advantage of being very strong, and, as a rule, thoroughly reliable.

(*d*) Silk may be used for either ligatures or sutures provided it has been thoroughly antisepticised by being kept for at least twenty-four hours in strong carbolic before being used. Silk may be further purified by being boiled, or sterilised by steam in a steriliser for half an hour, and then soaked in 1 in 20 carbolic till wanted. When perfectly aseptic, Lister has shown that it can be absorbed by the tissues, although the process is very slow. As a rule it acts as a foreign body and ulcerates out in the discharges, hence no more than is absolutely necessary should be left in the wound.

(*e*) Horsehair is used for sutures only in wounds where there is little tension, or where it is desirable that no scar should be left by the stitches, for example, in face wounds. It is not readily absorbed, but is very easily removed without causing pain. Unfortunately, prolonged immersion in antiseptic fluids renders it very brittle, so that we have to rely chiefly on cleansing it just before it is required. Experience shows that this is usually quite sufficient.

(*f*) Silver wire is used on the other hand in wounds where there is considerable tension on the edges, and usually in the form of button sutures. The buttons, which are to prevent the wire cutting out through the skin, are oval pieces of sheet lead, with projecting wings on each side, and a small hole in the centre. The wire having been been passed through the skin with a stout "wire-needle," each end is passed through the hole in a button, and one end having been fixed by being twisted round the wings, the edges are approximated, and then the other end of the wire is fixed in the same way. Usually the edges of the wound between the deep sutures are united by superficial sutures of horsehair or catgut.

The buttons are kept in carbolic.

Each of these materials should be kept in a jar similar to that used for the drainage tubes, containing 1 in 20 carbolic, except the horsehair and silver wire, which may be kept dry. *Caution!*—Before the operation begins you should remove the stopper from each of these jars and dust the edges with a swab of wool to remove the dust and its accompanying germs which has been landing on them since last used. If you neglect this precaution, when you come to withdraw a ligature it will collect all this dangerous matter and convey it to your wound. Let me warn you, too, against allowing ligatures or sutures to touch your own or the clothes of another as you hand them up.

Needles.—These are of various sizes and shapes, some being straight, others curved, half curved, and so on. Special needles are used for silver wire, having a groove running from the eye to the blunt end, in which the wire lies. Some needles have two eyes, the thread being passed through one and then through the other, thus guarding against its coming out.

Needles should be kept in a wide shallow glass tray, five or six inches in diameter and one and a-half inch deep, with a metal lid, rather than in a pin cushion. The advantages are (1) that they can be kept cleaner; (2) they can be kept sharper; and (3) they are less likely to be lost.

Always see that your needle is securely threaded, and test the suture before giving it to the surgeon.

Tourniquets are used to arrest or prevent hemorrhage. Before applying a tourniquet you should first empty the limb of blood as thoroughly as possible. This may be done by simply elevating it for a few minutes, or better still by applying Esmark's elastic webbing from the distal extremity towards the trunk, thus driving the blood before you. Having done so you must apply the first turn of your tourniquet very quickly and firmly, so as at once to arrest all circulation through the limb. If you fail to do this you will only arrest the venous return, without interfering with the arterial supply, the result being engorgement rather than depletion of the part.

(*a*) Petit's Screw Tourniquet.—One or two points must be attended to in fitting up this instrument. (1) Make sure that the band is properly threaded into the brass plates. To ensure this it must pass twice through each outer division in the under plate; and not at all through the inner division. If properly threaded no brass is visible on the under surface of the instrument, while if wrongly done the inner bar on each side is seen. (2) Be careful before beginning to thread the tourniquet that the buckle is turned so that it will catch when placed on the limb. (3) Approximate the two plates before beginning to apply the tourniquet.

(*b*) Foulis' Tourniquet consists of a piece of strong india-rubber tubing, about two feet long, and furnished with a simple catch. The tube is stretched and passed once or twice round the limb, and then fixed into the catch.

(*c*) Esmark's Elastic Webbing was originally introduced by Professor Esmark to empty the limb of blood preparatory to applying his large tourniquet of indiarubber tubing. It is, however, often used as a tourniquet itself, several turns being put tightly round the limb a short distance above the seat of operation.

### Nursing Medals and Certificates.

THE SUAKIN MEDAL.

THE following is a list of Nursing Sisters who have been awarded medals and clasps for the Soudan Campaign, 1884-85. Only H.M. Sisters received this medal. The National Aid Sisters were not eligible, as they did not belong to "The Service."

| Rank | Name. | Whether in possession of Egypt (1882) or Suakin (1884) Medal. | Whether entitled to clasps inscribed | |
|---|---|---|---|---|
| At the time the decoration was earned. | | | Nile 1884-85. | Suakin 1885. |
| Nursing Sister | King, H. ... ...... | Egypt (1882) | Yes | ... |
| Nursing Sister | Yardley, A. C.... | No | Yes | ... |
| Nursing Sister | Jerrard, M. C ... | No | Yes | ... |
| Nursing Sister | Gray, J. A. ........ | Egypt (1882) | Yes | ... |
| Nursing Sister | Hart, S. F. ...... | No | Yes | ... |
| Nursing Sister | Barker, M. ...... | No | Yes | ... |
| Nursing Sister | Byham, L. ...... | No | ... | Yes |
| Nursing Sister | King, J. .......... | No | ... | Yes |
| Nursing Sister | Norman, H. C... | No | ... | Yes |
| Nursing Sister | Williams, R. ... | No | ... | Yes |
| Nursing Sister | Hind, A. ......... | No | ... | Yes |
| Nursing Sister | Parsons, L. ...... | No | ... | Yes |
| Nursing Sister | Wallace, J. M... | Egypt (1882) | ... | Yes |
| Nursing Sister | Cole, M. C. F. K. | No | ... | Yes |
| Nursing Sister | Brown, S. J. ... | No | ... | Yes |
| Nursing Sister | Irving, C. F. ... | No | ... | Yes |
| Nursing Sister | Burleigh, R. M. | No | ... | Yes |
| Superintending Sister......... | Ireland, S. ...... | No | ... | Yes |
| Nursing Sister | Wright, E. ...... | No | ... | Yes |
| — | King, H. .......... | Egypt (1882) | ... | Yes |

### Appointment.

ROYAL INFIRMARY, HULL.—Miss Annie L. Cox, the General Hospital, Bristol, has been elected lady superintendent. Miss Cox was trained at the Derby Infirmary, where she had charge of wards, and remained three years, and subsequently acted as charge nurse of a large female ward (34 beds) for eighteen months. For two and a-half years she belonged to the nursing institution, West Maling. Miss Cox has acted first as night superintendent, and latterly as assistant matron at Bristol General Hospital during the last fifteen months. We congratulate the Royal Infirmary Committee upon their choice, and Miss Cox upon her election to a post so well suited to her abilities and powers.

ENERGY.

THERE are times and seasons when we feel more cast down by the troubles and trials of life than at others, such as an illness which takes us from some pleasant or very necessary employment. And again, when we have been working very hard and overtaxing ourselves. At such times we gladly catch at whatever gives us a little ease and respite, and the danger is lest we lose our energy altogether, and sink into sloth and self-indulgence.

There is an old Greek story which is a good instance of this. Ulysses, the King of Ithaca, had taken his men to the siege of Troy, where they had fought valiantly for ten years. The war being over, they started for their sterile, rock-bound island with great joy, longing for home, and looking forward to meeting those dear relations from whom they had been so long parted. But, alas, many misfortunes befel them, they were disheartened by the strange temptations and unheard-of dangers which beset them on all sides, and in which but for their wise leader they would have miserably perished. By obeying his orders they were led safely through all, until they were shipwrecked on a lovely and enchanted island. There everything invited them to ease and pleasure. Lovely flowers, luscious fruits, murmuring streams, and the gentle rustle of the soft winds among the trees, wooed them to sleep and self-indulgence. The poet says "It was a land in which it seemed always afternoon," and nothing can express more forcibly the charm of that part of the day when, our work being over, we can rest and be thankful. So thought these old warriors; they were so taken up by the pleasures of the moment, so happy in these seductions of their senses, that they forgot their homes and all that had formerly been so dear to them, "Fatherland and wife and child and slave," and wanted to remain where they could eat and drink their fill and doze and indulge every selfish wish.

We often get tired of striving and struggling and think it is true happiness to do nothing but what pleases us. Don't let us give way to such wishes. Work is the best and greatest good we can have, it takes our thoughts off our misfortunes if we have any, and helps us to that energy without which we are worthless. If those Greeks had been told, when they were setting out with their king, what would have been their ending, they would have scoffed and said with the king of Israel, "Is thy servant a dog that he should do such things?"

We Christians have a King who will help us to stand up against pleasant sins. We must, however, work with Him, we must wish and will, with all our hearts, not to give way to sloth, and we shall be set at last by Him who is mighty to save in that glorious land where the flowers never fade, and the Tree of Life "bares twelve manner of fruits and yields them once a month," where the praise of God is as the sound of many waters, where God shall wipe away all tears from all eyes, and where there is no more death, nor sorrow, nor crying, nor pain. He that overcometh shall inherit all these things.

### American News.

New York, Jan. 25th.

The treatment of the insane is the burning question in hospital circles here. Miss Clarissa C. Lathrop, who was incarcerated for 26 months in an asylum in Utica, is forming an Anti-Kidnapping and Lunacy Reform Union.

The object of the Union is to prevent the detention of sane persons in insane hospitals, and to procure legislation for bettering the condition of the insane. The President is Bronson Murray, of New York, the Secretary Miss Lathrop, the Treasurer Madam Demarest. Miss Lathrop intends to present to the next Legislature a Bill to prevent the detention of sane persons in insane hospitals. One of the provisions is that each inmate shall be allowed to have at least one correspondent of his or her choosing to whom he or she may write and personally mail the letters so that they may not be apprehended or read by the asylum authorities. That free visitation by friends is a necessity to recovery, Miss Lathrop earnestly believes. She entirely differs from the State Commissioners in Lunacy, who, in a letter to the Erie County Board of Supervisors a short time ago, expressed their opinion that removal from the locality of home, and infrequent visits by friends, were more conducive to restoration of mental health. Before the Woman's Legal Education Society of New York, Mrs. Emily Kempin yesterday delivered a lecture on "The Rights of the Insane." Mrs. Kempin said the laws regarding the insane were most unsystematic and illogical, not only in the States, but all over the world. The lunatic loses his personal and social liberty and his moral existence; he loses more than the criminal, and yet he loses it solely on the word of two physicians. His fate is settled, not in public, but within the four walls of a consulting-room. With regard to this question, it is notable that James A. Woods, attendant at the Easton Hospital for the Insane, was this week sentenced to 21 years' imprisonment for kicking a patient to death.

*The Nightingale* prospers, and becomes more interesting every week. In a late number was an account of how the nurses of St. Paul's Home, Rome, were received by Lady Dufferin on Christmas Day and provided with tea. The Earl was also present, and chatted to the nurses.

The Society of Medical Jurisprudence has just held its annual dinner, which is always an amusing affair. The doctors and the lawyers love to make jokes at one another's expense, as may be judged from a toast list which contained the following: "The Tribulations of Experts," "The Victims," &c.

The Wisconsin Training School for Nurses is in connection with the National Soldiers' Homes, and graduates about seven nurses annually. One of its members, Mrs. Darling, has been appointed Matron of the hospital at Oconto. It is the only training school in the State, and its private nurses are in great demand. On Christmas Day the Home received two presents, a piano and a gift of land. We are very great on societies over here. The graduates of nearly every school have formed themselves into associations, and meet monthly and read papers to one another. The American Nurses' Association, which is on a wider basis, does not seem so popular. The Guild of St. Barnabas does very well. The Committee have lately draughted a constitution which is being submitted to the various branches. A special service and sermon in favour of the Guild were held at the Church of the Holy Communion on January 22nd.

Amongst our many hospitals chiefly supported by ladies, and blessed with pretty names, is "The Flower Hospital," free to surgical cases. In aid of this institution an appropriate entertainment has been given, in which about thirty New York belles went through the evolutions of the "Milkmaid's March." The scene was a charming one, and a nice sum was secured for the hospital.

The following quotation is interesting as showing the similar line taken in England and here by the best nurses: "To the Nightingale.—There has been some talk about a registry for nurses in connection with the Academy of Medicine whether we want it or not. Now the genuine New York nurse doesn't need this special registry and doesn't particularly want to pay the additional fee. Graduates of Mt. Sinai, of New York Hospital, of Bellevue, and of the Post-graduate don't need a registry. The work coming through their hospitals is sufficient to keep all reputable graduates employed."

### The Nurses' Bookshelf.

### LECTURES ON NURSING.*

Here we have two volumes of lectures delivered for the St. John Ambulance Association, and both bearing a strong resemblance to former lectures published by former lecturers of the society. Indeed, we cannot but repeat that we think it a pity that so many of these books are printed; their only *raison d'être* can be to be sold to the students of the classes at which the lectures are delivered. The St. John's Association might with advantage publish with its authority one of the best of these numerous volumes and save us from the others. But in one direction the two books before us are an advance on their predecessors—they are both illustrated, and they both give specimen charts. In truth, both books are well written and useful, containing all the usual information, and concluding with an appendix. Dr. Lawton Roberts gives the rules and objects of the nursing guilds or corps which have been formed at Guernsey and elsewhere by students of the St. John Ambulance Association; Dr. Fitzgerald has given considerable attention to the wider subjects of personal and family hygiene.

### WOMAN'S HEALTH.†

Day by day we become more convinced of the fact that there ought to be more lady doctors and more male nurses; and a forcible argument in favour of lady doctors is to be found in Dr. Rentoul's remarks on woman's health. The subject is approached only from the masculine point of view, and a woman is regarded solely as a mother of children. From the age of twelve a girl is to spend three months of every year flat on her back; she is not to study, she is not to play, she is not to plunge her hands and arms into cold water, she is to acknowledge her unfitness for continuous work. This is Dr. Rentoul's view, and it properly belongs to the eighteenth century — that period which has so rightly exercised the wrath of Carlyle. On page 49 Dr. Rentoul has some very sensible remarks on the force of habit, and we would put it to him that it is only when a girl of twelve forms these habits of unnecessary carefulness that they become essential for the maintenance of health. A nurse can do her day's work from year's end to year's end, and take her cold tub every morning; and the ranks of school-mistresses, seamstresses, type-writers, &c., show that sustained mental and bodily exertion is possible to the average woman. The upper classes, who need not work, are not exempt from the diseases of women; in fact, it is at the door of the idler the doctor's brougham most often stops. If Dr. Rentoul could for one moment remember that a woman has a mind as well as a body, he might be brought to acknowledge that to lie

* "Physiology and Hygiene for Home Nursing," by C. E. Fitzgerald, M.D. (London: Messrs. Bell and Sons.)—"Nursing and Hygiene," by R. Lawton Roberts, M.D. (London: Mr. H. K. Lewis.) Price 2s. 6d.

† "The Dignity of Woman's Health," by R. R. Rentoul. (London: Messrs. J. and A. Churchill). Price 3s. 6d.

on a sofa and make a fuss over natural functions, and give far too much thought to one's internal economy, is the very way to induce the morbid conditions which lead to disease. But, then, Dr. Rentoul has no faith in nature; witness this amusing sentence: "Thus the designs of Providence are knocked out of time, and so women are left husbandless." Perhaps Providence can look after its own designs, and they might possibly include spinsters. To approach the subject of woman's health as though she were merely an animal is to betray the absence of higher feeling in the author, rather than in the beings he maligns.

### PERSONAL ANECDOTES.*

This is a chatty and amusing little book, but decidedly childish, and very badly put together. Sister Eva began nursing at the age of eighteen, and is now only 24; her portrait graces the commencement of the volume, though we missed it and the introduction for some time, owing to the way in which the advertisements are bound up in the matter.

Sister Eva commenced her experiences under the charge of a sisterhood, and they made her black the grates, and punished her by giving her extra work when she used the tenax to light the fires. Apparently the unbecoming garb of the sisterhood was too much for the would-be nurse, for her after-experiences seem to be in connection with some institution. The chapters of the book are utterly disjointed, and the matter has no sequence; yet the authoress is capable of dramatic writing when she chooses, as the incident of "The Miser" shows. In keenness of observation Sister Eva is evidently excellent, therefore it is the greater pity that she misses the great nursing virtue of method. At some future day we shall expect to review a much better book from her pen; meanwhile we can promise our readers there is half-an-hour's amusement to be had in perusing these "Scenes."

* "Scenes in the Life of a Nurse," by Sister Eva. (London: Messrs. Bemrose and Sons.) Price 2s.

### Everybody's Opinion.

### THE DINNER HOUR.

"D. C." writes: There has been much discussion lately about nurses and their hours of work, leisure, and rest. Will you allow me to make the following suggestion to those in authority. Every class of workers from high to low has what is called the "dinner hour"; there is one exception only—nurses have no "dinner hour." A nurse's dinner is taken in the middle of a busy day; she has generally a half-hour allowed for it. She rushes off to it, fearful of being late, leaving behind her a crowd of duties. Dressers and house-surgeons just going away from the ward, leaving behind them a great deal of work, which every nurse who has been her time in a surgical ward will know what it means to get over, leaving, at the same time, everything ready for the next time it is wanted. Patients' dinners are just coming up or in full swing; the time is the very busiest in the day; yet to dinner she must go, leaving all behind her, or, rather, taking it with her, to think about, and wonder how she is to get it done when she gets back, so as to be able to go off duty at two, when the Sister tells her. Dinner hastily swallowed, or often only looked at, the nurse has to hasten back and relieve the one on duty, so she can go through the same pretence of dinner-eating in her turn. The one who comes back to the ward finds some of the work done, for everything must be ready for the staff, who will soon be coming round. Now, would not it be more reasonable for nurses to have their dinner at a time when they could have a chance of eating it comfortably and at leisure, when they could have really a "dinner-*hour*"?—a most important item as regards health, digestion, and temper, for I believe that indigestion is the fox which is spoiling the vines, and creating discontent and unhappiness in our hospitals. But now let me suggest a plan. From four to six is a time of comparative leisure in hospital work; there is only tea to think about till evening work begins, unless, of course, there is something extra to do, but that is not every day. At any rate, each nurse could have then her "dinner-hour," and not be missed.

### Princess Christian's Daughter.

MISS E. DURHAM, Farringford, Freshwater, Isle of Wight, acknowledges the following additional subscriptions towards a wedding present for Princess Louise of Schleswig-Holstein to be given as a proof of the gratitude of nurses for the interest Princess Christian has ever taken in their progress. Subscriptions will be received until the end of March, and will be acknowledged in these pages. From February 9th to February 16th the following sums were received:—

*Matrons* (each 5s.)—C. Doran, S.W. *Sisters* (each 2s. 6d.)—A. M. S., Ruth Cumberland, Alice Goodwin; Nurse Robertson, Nurse Roach, Nurse Bingham (each 2s.) *Nurses* (each 1s.)—Clara Porter, Sarah Arnold, Louise Nelson, Nurse Slaughter, L. E. Stone, C. Carter, Nurse Dare, B.N.A., Sarah Clark, B.N.A., Selina Moody, B.N.A., Mary Fuller, A. Gordon Farrer, S. A. Pennington, S. A. Anthony, J. Dolling, Annie Alloway, Nurse Juliet, Annie Bridle, Nurse White, Nurse Murray, Nurse Waite, and Alice Montgomery.

### Presentations.

GRAVESEND HOSPITAL.—A very pleasing presentation was made at a social gathering of the nurses at this hospital last Thursday week. The nurses desired Mr. Woodhams, the House Surgeon, to present to the Matron (Miss Walker) a handsome solid silver card case as a token of their esteem and regard. In making this gratifying presentation, Mr. Woodhams alluded in very appropriate terms to the kind feeling existing between the Matron and her nurses. The presentation had been kept an entire secret from the Matron and was a very agreeable surprise to her. A very happy evening was spent.

MISS YOUNG, who is leaving Addenbrooke, has been presented with a silver tea-caddy by the physicians, with a clock by the House Physician, and with brass candlesticks, writing set, and handsome screen by the nursing staff. The Committee have recorded in the minutes their regret at Miss Young's departure.

ON February 12th, Miss Ransford, for five years matron of Noble's Isle of Man Hospital, was presented with an album and a purse of sovereigns. The presentation was made by his Excellency the Governor, who was supported by the Lord Bishop and Deemster, Sir W. L. Drinkwater. There was a large gathering of visitors, besides the medical staff, nursing staff, servants, and a few of the patients. General regret was expressed at Miss Ransford's departure, and great credit given her for the work she had done at the hospital.

### Notes and Queries.

### Answers.

(34) Poultices back and front are most comfortable when made on old linen, shaped at neck and arms; stitch tapes to the sides, and pin at shoulder with safety pin; over-pin a small towel (this keeps the poultice warm, and gives the patient less fatigue than bandaging).—*S. Hall, Rotherham.*——Make a short flannel jacket, buttoning up both front and back: this will keep the poultice in place, and permit you to change it rapidly.—*V. M.*

*J. R. D.*—It refers to a presentation in which the First Thousand only are interested. Full particulars will appear here later.

*M. T.*—We have no room to print your lines.

*Matrona.*—The usual allowance is three to eight private nurses to a bed; the smaller number for comfort, especially at the beginning. Experience will soon prove whether your institution is popular enough to risk the larger number.

*L. S.*—We have no room to print your paper.

*Probationers.*—Our correspondents who desire to enter hospitals as probationers are requested to get "The Hospital Annual;" there they will find particulars we have no room to keep on repeating here.

*A Nurse.*—Brush the hair well every night, wash it weekly in one in forty carbolic. These as precautions, the remedies you mention if thoroughly used would be effectual. Please write on one side of the paper only.

*Enquirer.*—You can only advertise in our pages or in the *Lancet* for posts in the English Colonies; or you can write to the Agent-General of any particular colony. THE HOSPITAL circulates very widely in Australia. Your letter appeared last week: we often have to hold over letters in order to get in news up to date.

### Nurse Hilary.

(*Continued from page cxii.*)

THE life or death of a fellow-being lay as it were in Nurse Hilary's hands. There must be no hesitation, no shirking, on her part. She must be "on duty." Alan was a soldier now; *he* would know what that meant! and yet—it was hard! Alan's face came before her, the well loved kindly brown face she might never see again. Her breath caught in two or three dry gasping sobs. Then she pulled herself together, and in another moment she was standing by bed No. 10, quiet, self-possessed, with her mind concentrated by resolute will on the injured woman who lay there. She was a small fragile woman with a worn, yet somewhat childishly pretty face; the petulant and weak mouth, and fluffy fair hair adding to the impression. She lay quite still and unconscious, her left hand with its broad wedding ring resting on the scarlet quilt. Hilary put a sheltering screen round the bed and the ward became very quiet.

The little doctor came in presently, giving her an approving nod and smile as he bent over the helpless figure.

"I must operate at once," he said, shortly. "It is a good thing you stayed." All through the next hour Nurse Hilary stood watchful, alert, ready, every faculty strained whilst the skilful, critical work went on; and when it was done and the woman given over to her complete care, she still stood by her with every nerve strung to its tensest pitch, waiting for the return of consciousness. It came at last. The blue dazed eyes opened, and she broke into low wandering words:

"Alan! Alan! Come back! I am so lonely. Why did you go away? Oh, do come! I want you so."

Tears which she had driven back before swept into Nurse Hilary's eyes. Someone else had an Alan, too! Someone else wanted him!

"I am so lonely now, so lonely. Mother went, too—somewhere. I lost her in a shop or"——

"Hush!" said Nurse Hilary quietly. "Drink this."

The woman looked at her with a feeble far-off look, then held up her hand. "Do you hear the rain? Alan will be so wet. It is so dreary in the rain—travelling, too." She clutched the scarlet quilt. "Take him this, please. Tell him to put it on—he knows I *do* care, but we were often unhappy, weren't we? Mother would be so sorry, but I mustn't tell her, Alan would be angry. I must run after him." She tried to raise herself, but Nurse Hilary held her.

"Alan! Alan! Alan! I *cannot* find you. Where are you, dear?" She sank back exhausted, and closed her eyes; then she began, in a quieter voice, again—"I had a photo somewhere. I want it. I want to see it. Can't you find it?" She groped about restlessly with her hand.

There was a little handbag lying on the locker. Hilary opened it to see if she could find the photograph to quiet the poor dazed mind. A bunch of keys, a handkerchief, an old veil formed its contents, and underneath lay a photograph, face downwards. She took it out, and looked at it.

Was it a second's space or an eternity? She felt as though she had become in a breath a woman of stone, petrified, numb, cold, colder than winter's ice.

It was Alan's face, Alan's figure she held on this bit of card. Alan, as she had never seen him, in his private's uniform. *Her Alan*! Upright, smiling, looking straight out at her!

A great shiver passed over her. She knew vaguely that a sword had been struck deep into her heart, and yet she could not feel it. She turned and mechanically put the photo into the woman's hand; mechanically, too, she gently laid down the restless figure, smoothed the pillow, and pulled the quilt higher.

Then she sat down by the bed and found herself reiterating in her inner conciousness. "It is not true—it is all a mistake, I cannot believe it, I cannot—I cannot."

"It is just like him" went on the rambling voice near her; "he was so good to me—so good—" and the woman fell into a restless sleep, holding the photo tightly clapsed in her hand.

The chimes of some church near tolled out six o'clock. Every stroke fell like the notes of a loved one's passing bell on Hilary's heart; and yet she kept faithful to her post, watching the wavering life, feeding, soothing with unceasing care hour after hour, till at last the broken delirious murmur ceased, the wandering eyes closed, and the woman dropped gently into a quiet, natural sleep. Late in the evening she awoke, and Hilary saw that her eyes were clear and conscious.

"Where am I?" she asked feebly. "Was I hurt? My husband won't know." She looked anxiously round her. Hilary bent over her—she must know the truth and now was the moment.

She said quietly and distinctly, "What is your husband's name?"

"Alan Webster."

It seemed to Hilary as if a great mental darkness fell upon her, and that the last golden spark of her life's best hope died as it fell.

As if in a dream, she saw the little doctor appear, saw his quick comprehensive look at the patient, and heard his words of approval.

"She'll do now, thanks to you, nurse. Capital! Capital! We'll soon set you right again; you hurt your head a bit, you know, but it's all over now."

"Yes," said Hilary to herself, "it is all over, and for ever."

(*To be continued.*)

### Amusements and Relaxation.

### SPECIAL NOTICE TO CORRESPONDENTS.

First quarterly word competition commenced January 3rd, 1891; ends March 28th, 1891.

Competitors can enter for *all* quarterly competitions, but no competitor can take more than *one first prize* or *two* prizes of any kind during the year.

Proper names, abbreviations, foreign words, words of less than four letters, and repetitions are barred; plurals, and past and present participles of verbs, are allowed. Nuttall's *Standard* dictionary only to be used.

N.B.—Word dissections must be sent in WEEKLY not later than the first post on Thursday to the *Prize Editor*, 140, Strand, W.C., arranged alphabetically, with correct total affixed.

The word for dissection for this, the EIGHTH week of the quarter being "HYACINTH."

| Names. | Feb. 5th. | Totals. | Names. | Feb. 5th. | Totals. |
|---|---|---|---|---|---|
| Reynard | — | 77 | Woodbine | — | 25 |
| Reldas | 67 | 299 | Madame B. | — | 25 |
| Tinie | — | 30 | Shakespeare | — | 59 |
| Patience | — | 76 | Smyrna | 57 | 186 |
| Jenny Wren | 55 | 259 | Southwood | — | 102 |
| Agamemnon | 68 | 291 | Gipsy Queen | — | 21 |
| Wyamaris | 61 | 284 | Snowball | — | 19 |
| E. C. | 67 | 295 | Rita | — | 88 |
| Ecila | 61 | 283 | Mortal | — | 16 |
| Hope | 69 | 295 | Nurse Annie | — | 15 |
| M. W. | 64 | 291 | Carmen | — | 11 |
| Qu'appelle | 64 | 289 | Grannie | — | 45 |
| Nil Desperandum | 70 | 293 | Amie | — | 30 |
| Lady Betty | 60 | 274 | M. R. | — | 25 |
| H. A. S. | 56 | 239 | Primrose | — | 24 |
| Sister Jack | — | 62 | Nurse J. S. | 62 | 100 |
| Crystal | 47 | 203 | B. A. C. | 43 | 43 |

## Figures and Tables

**Figure f1:**
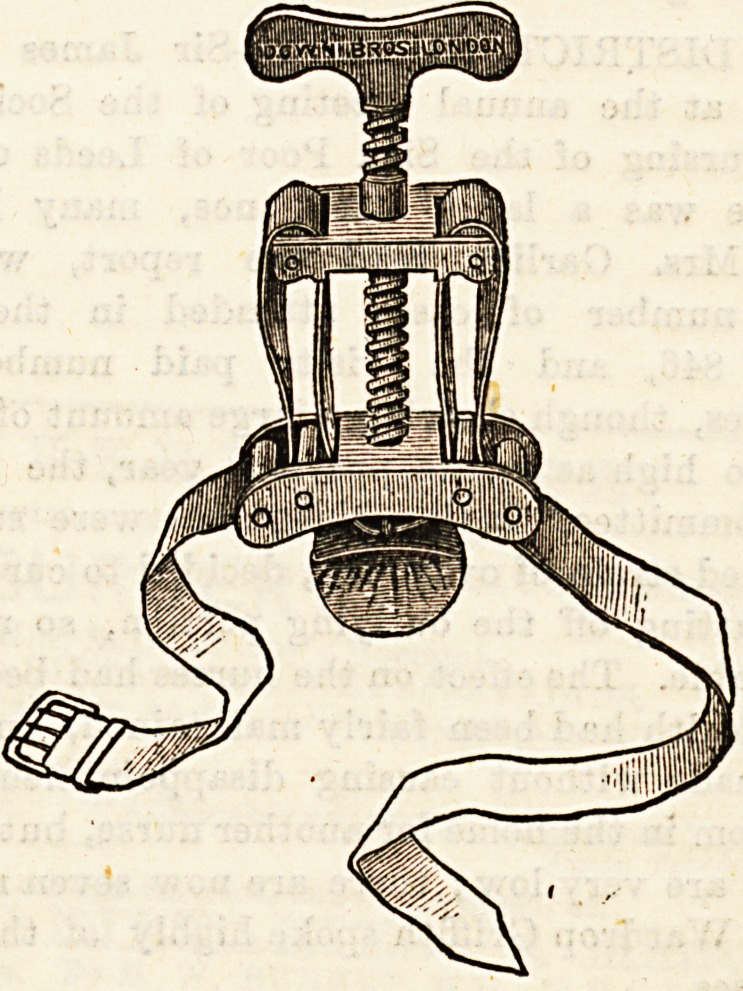


**Figure f2:**
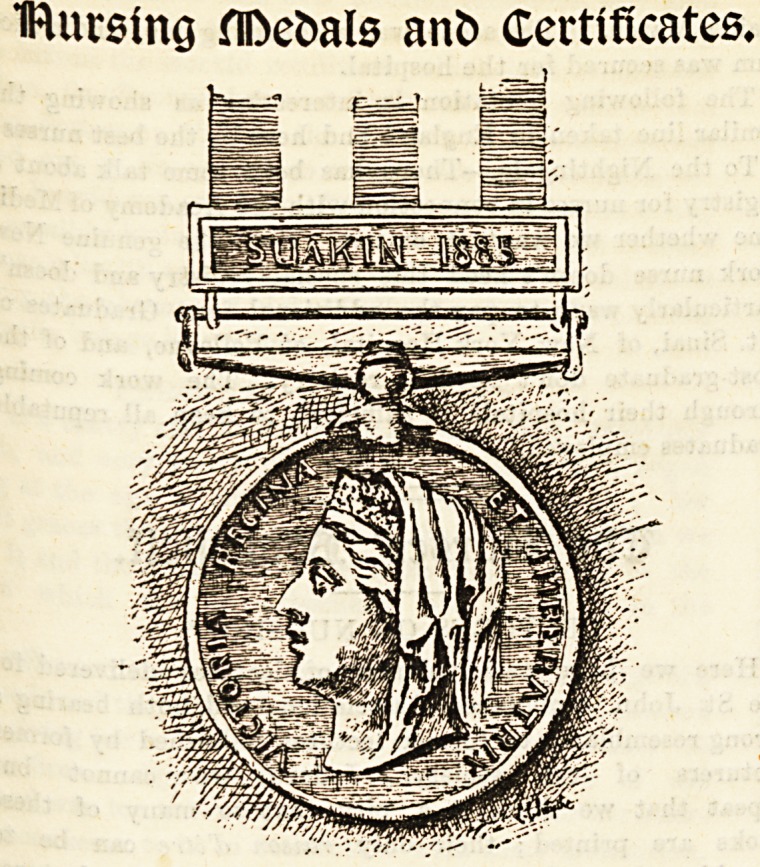


**Figure f3:**